# FMG- and RNN-Based Estimation of Motor Intention of Upper-Limb Motion in Human-Robot Collaboration

**DOI:** 10.3389/frobt.2020.573096

**Published:** 2020-12-03

**Authors:** Mohammad Anvaripour, Mahta Khoshnam, Carlo Menon, Mehrdad Saif

**Affiliations:** ^1^Department of Electrical and Computer Engineering, University of Windsor, Windsor, ON, Canada; ^2^Menrva Research Group, Schools of Mechatronic System and Engineering Science, Simon Fraser University, Vancouver, BC, Canada

**Keywords:** human-robot collaboration, collision avoidance, recurrent neural network, force myography, industrial robot

## Abstract

Research on human-robot interactions has been driven by the increasing employment of robotic manipulators in manufacturing and production. Toward developing more effective human-robot collaboration during shared tasks, this paper proposes an interaction scheme by employing machine learning algorithms to interpret biosignals acquired from the human user and accordingly planning the robot reaction. More specifically, a force myography (FMG) band was wrapped around the user's forearm and was used to collect information about muscle contractions during a set of collaborative tasks between the user and an industrial robot. A recurrent neural network model was trained to estimate the user's hand movement pattern based on the collected FMG data to determine whether the performed motion was random or intended as part of the predefined collaborative tasks. Experimental evaluation during two practical collaboration scenarios demonstrated that the trained model could successfully estimate the category of hand motion, i.e., intended or random, such that the robot either assisted with performing the task or changed its course of action to avoid collision. Furthermore, proximity sensors were mounted on the robotic arm to investigate if monitoring the distance between the user and the robot had an effect on the outcome of the collaborative effort. While further investigation is required to rigorously establish the safety of the human worker, this study demonstrates the potential of FMG-based wearable technologies to enhance human-robot collaboration in industrial settings.

## 1. Introduction

Incorporating robotic technology in the industrial environment has facilitated the manufacturing process by increasing flexibility and productivity (Finkemeyer and Kiel, 2017). While the robot or the human might separately handle their given tasks, in some cases, sharing the workload increases the quality and productivity while avoiding excessive fatigue for human workers (Bi et al., 2019). Any collaborative scenario should put into place strategies to ensure safety of workers, intuitive interfaces to establish clear communication between the human and robot, implement control schemes, and deploy sensor network for task coordination and planning the trajectory of the robot (Villani et al., 2018).

Toward avoiding collisions and preserving safety of workers while promoting productivity, the human-robot communication during a shared task might be enhanced by implementing means to detect the presence of humans in close proximity of the robot and developing a scheme in which robot reactions are adjusted in accordance with an estimation of human intentions for their next move (Avanzini et al., 2014; Bi et al., 2019).

In shared workspaces, vision-based systems have been used to monitor the dynamic location of humans, objects, and robots (Halme et al., 2018). Using the acquired images, a variety of algorithms have been proposed to monitor the distance between the human and the robot, ensure collision avoidance, estimate human motion pattern, and recognize gestures to facilitate human-robot communication (Pérez et al., 2016; Halme et al., 2018; Liu and Wang, 2018). The effectiveness of vision-based systems have been demonstrated in different simulated industrial environments, however, challenges, such as computational complexity, performance degradation as a result of dust or poor illumination, and the risk of occlusion still limit their efficacy in real-time estimation of human intentions and subsequent planning of the robot trajectory to avoid collisions (Avanzini et al., 2014; Pérez et al., 2016; Halme et al., 2018). Depending on the application and purpose, augmentative or alternative to the vision-based systems could be instrumenting the robotic manipulator with distance sensors (Avanzini et al., 2014; Halme et al., 2018) and/or taking advantage of wearable technologies (Liu and Wang, 2018; Bi et al., 2019). Wearable devices, e.g., in the form of gloves and bands, are growing non-image-based technologies for gesture recognition in human-robot interactions that can provide fast responses and can be used to incorporate an estimation of human intentions when planning the robot trajectory (Liu and Wang, 2018; Bi et al., 2019).

Incorporating human intentions in planning the robot trajectories and reactions increases the flexibility and safety of cooperation (Bi et al., 2019). While various image-based communication methods, such as gaze tracking (Sakita et al., 2004; Zhao et al., 2012), have been proposed, other sources of information have been investigated as well (Bi et al., 2019). Approaches that use data from sources, such as surface Electromyography (sEMG) electrodes and inertial measurement units (IMUs) are examples of the latter category (Assad et al., 2013; Chen et al., 2017; Wang et al., 2018; Bi et al., 2019). In such cases, upper-limb movements are observed to estimate if arm and hand motions are aimed at collaborating with the robot or are random (Bi et al., 2019). Probabilistic models, e.g., machine learning algorithms that do not require a complete model of human behavior, have been employed to process the data collected by sensors. Hidden Markov model and neural networks are examples of methods used for estimating human intentions in collaboration with a robot (Wang et al., 2009; Ge et al., 2011; Ravichandar and Dani, 2017; Schydlo et al., 2018).

Force Myography (FMG) is a technique to quantify changes in the volume of a limb resulting from muscle contractions and relaxations (Xiao and Menon, 2014). This biosignal has been employed in a variety of applications including gesture recognition, control of exoskeletons, prostheses, and linear actuators, and estimation of user-applied forces to manipulate planar linear actuators (Xiao et al., 2014; Cho et al., 2016; Sakr and Menon, 2016a,b, 2017, 2018; Jiang et al., 2017; Sadarangani and Menon, 2017; Zakia and Menon, 2020). Force myography from upper-limbs could be collected using lightweight, compact, and unobtrusive bands wrapped around wrist, forearm, and/or upper arm, which makes it an attractive technique for developing wearables.

We have previously shown that the support vector machine (SVM) model trained with two features extracted from FMG data, namely power spectral density and likelihood, could classify six different hand gestures with an accuracy of above 90% (Anvaripour and Saif, 2018b). We also demonstrated that FMG data could be used to estimate the forearm muscle stiffness. Such an estimation was then applied to adjust the robot gripper force to handle different objects with the same gripping force as that of the human worker (Anvaripour and Saif, 2018a). Further, we showed that the information provided with the FMG band along with the robot dynamics can be used to plan the trajectory of the robot during a shared task. The proposed approach was tested in a scenario in which the robot and the human worked together to carry a shared load along a predefined trajectory (Anvaripour et al., 2019). This paper builds upon our previous works to incorporate an estimation of human intentions to improve work flow during performing the shared task, i.e., the task continues without interruptions when the human is performing movements required to complete the task. To this end, an FMG band was placed around the forearm to record changes in the muscle volume. A recurrent neural network (RNN) with Long Short Term Memory (LSTM) architecture was implemented to estimate human intentions based on multiple features extracted from the collected FMG data and the robot dynamics. Moreover, this study takes our previous investigations a step further by using the information collected with proximal sensors mounted on the robot arm to plan and execute evasive motions to prevent a collision when the human is in the proximity of the robot. The proposed approach was successfully tested in two practical scenarios in which a human and a robot worked collaboratively to complete defined shared tasks. Although this method requires an *ad-hoc* sensory system for each individual, noting that FMG is a relatively inexpensive technology, the proposed approach does not considerably increase the hardware or computational cost. Therefore, such a method can be used as augmentative to the more established image-based methods, for example to compensate for an obstructed view or to enhance the real-time estimation of human intentions and planning of the robot trajectory.

## 2. Collection and Processing of FMG

In this section, the designed FMG band used to monitor volumetric changes in the human forearm muscles is introduced. The signal processing technique and the feature extraction method to acquire information about human forearm muscle contractions/relaxations are explained. The resulting features are then used to estimate the force that the human applies on the robotic manipulator (section 2.3), control the robot (section 3), and train the proposed neural network to estimate human intention during the collaboration with the robot (section 4.1).

### 2.1. Forearm FMG Band

**Figure 1:**
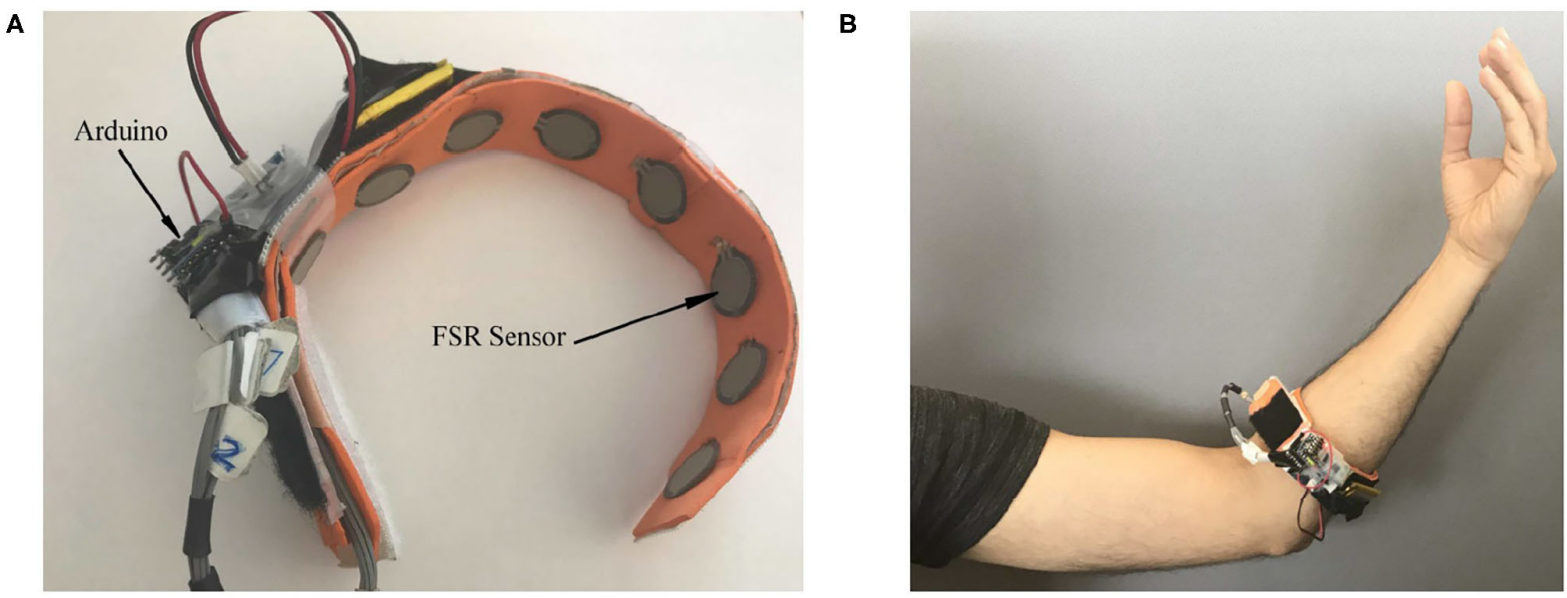
**(A)** The FMG band equipped with FSRs, **(B)** positioning of the band around the forearm. (Reproduced with permission).

Force myography data can be collected with force sensing resistors (FSRs), the output of each sensor depends on the amount of force applied to the active area. For this study, a custom FMG-band with eight FSRs (FSR 400, Interlink Electronics, Inc., Los Angeles, CA) was used. The band was wrapped around the forearm muscle as shown in Figure 1 such that FSRs were in contact with the user's skin. Signals from FSRs integrated in the band would then indicate muscle contraction patterns resulting from changes in the volume of the forearm muscles while performing a manual task. To make the band wireless and more compact, a microprocessor (ATMega328, Microchip Technology, Chandler, AZ) was programmed to collect data from each FSR and transmit them to an on-site computer through a Bluetooth module (HC-05 Wireless Ibeacon Module) at a sampling frequency of 25 Hz.

### 2.2. Forearm Muscles Contraction Patterns Graph

Data collected from FSRs were processed using a sliding window. Denoting the sampling frequency of the FMG band with *f*_*s*_, a total of 1/*f*_*s*_ samples were extracted in 1 s. A window of size *w* > 1/*f*_*s*_ was applied to extract the features. The sliding window was moved in time by *m* samples where *m* < *w*; thus, every two consecutive windows had an overlap of the size of *w*-*m* samples. Signal segments in each time window were debiased by calculating each segment's mean value and subtracting it from the signal values in the corresponding time frame. Power spectral density (PSD), one of the features commonly used for the training of machine learning algorithms, was then calculated for each time window (Saa and Gutierrez, 2010)

(1) $$\begin{array}{l} \begin{array}{c} PSD=\frac{|X\left[k\right]{|}^{2}}{f_{s}.w}\\ X\left[k\right]=\overset{N-1}{\underset{m=0}{\sum }}x_{w}e^{\left(-2\pi i\left(m-1\right)\right)/N},\;k=0,1,...,N-1 \end{array} \end{array}$$

where *N* is the length of the frequency window used for Fast Fourier Transform (FFT), *f*_*s*_ is the sampling frequency, *w* is the window length, *m* determines the number of overlapping samples in two consecutive time windows, *X* is the frequency domain representation of the collected signal, and *x*_*w*_ is the segment of collected signal corresponding to window *w*.

Likelihood was selected as another feature and calculated as

(2) $$\begin{array}{l} l_{n}=\frac{k_{s}\times M_{s}}{1+M_{s}}-\text{log}\left(1+M_{s}\right) \end{array}$$

where $k_{s}=|X{|}^{2}/\text{var}\left(N_{s}\right)$ is the *a posteriori* signal to noise ratio (SNR). The variance of noise for calculating this parameter can be obtained in the training phase of the model. The *M*_*s*_ is the *a priori* SNR calculated using the decision-directed method (Ephraim and Malah, 1984).

The feature extracted from each window was a combination of the above features:

(3) $$\begin{array}{l} Q\left(n\right)=\overset{n}{\underset{i=n-w_{s}}{\sum }}\alpha_{i}{PSD}_{i}.e^{\beta_{i}l_{i}} \end{array}$$

where α and β parameters could be determined experimentally.

Since different arm and hand movements activate different muscles, data collected during various arm and hand movements/gestures are distinct, and extracting the relationship between them creates a unique description of the performed. To define such a relationship quantifying of the forearm muscle, data from all sensors were considered together to form a graph of *m* sensors at each time step:

(4) $$\begin{array}{l} Y=\left[\begin{array}{cccc} 0 & \left|Q_{2}-Q_{1}\right| & ... & \left|Q_{m}-Q_{1}\right|\\ \left|Q_{1}-Q_{2}\right| & 0 & ... & \left|Q_{m}-Q_{2}\right|\\ \vdots & \vdots & \vdots & \vdots \\ \left|Q_{1}-Q_{m}\right| & \left|Q_{2}-Q_{m}\right| & ... & 0 \end{array}\right] \end{array}$$

This matrix is symmetric, i.e., *Y*(*i, j*) = *Y*(*j, i*), and can be interpreted as a graph with *m* vertices and $\frac{m^{2}-m}{2}$ distinct edges which represent the relationship between each two sensors.

### 2.3. Estimating Forces Applied on the Robot

The measured graph *Y* can be normalized using the maximal voluntary contraction (MVC) feature denoted by *V*. This value refers to the maximum external force that the robot arm can tolerate during the cooperation. *V* is determined experimentally for each specific application. The normalized matrix describes muscle FMG and is obtained as

(5) $$\begin{array}{l} R_{i,j}=\frac{Y_{i,j}}{V_{i,j}} \end{array}$$

where 0 ≤ *R* ≤ 1. The activation level for each sensor is defined as

(6) $$\begin{array}{l} D=\overset{m}{\underset{k=1}{\sum }}R\left(:,k\right) \end{array}$$

where *m* is the number of force sensors used in the FMG band, and *D* is a diagonal matrix in which the elements are the sum of the associated activation level of each sensor used to capture the FMG. To find the corresponding value of force for use in the robot controller, the following mapping is defined

(7) $$\begin{array}{l} f_{r}=c\left(f_{max}-f_{min}\right)+f_{min} \end{array}$$

where *f*_*min*_ and *f*_*max*_ are the minimum and maximum controllable force externally applied to the robot. Anvaripour and Saif (2018a) were defined the mapping parameter *c* as follow

(8) $$\begin{array}{l} c=T_{1}\frac{1-e^{T_{2}DD^{T}}}{1+e^{T_{2}DD^{T}}} \end{array}$$

where *T*_1_ and *T*_2_ are mapping parameters obtained experimentally.

The translated applied force value can be used to estimate human intentions, e.g., whether the user intends to move the manipulator as part of a pre-defined collaborative task. Consequently, it can be incorporated in the robot control algorithm to adjust the endpoint velocity and joints torques during the cooperation such that the robot reacts accordingly to estimated human intentions.

## 3. Robot Control Algorithm

The system under study is a robotic platform directly interacting with or working in proximity of a human. In such cases, user's hand is the most vulnerable limb to injury if there is a collision with the robot arm during a collaborative scenario. Considering that the location of the object that the robot is working on as well as the endpoint trajectory are both defined in the control algorithm, describing human limb motions with respect to the same coordinates and estimating human intentions facilitate adjusting the controller parameters.

The robot dynamic can be presented by a second order equation:

(9) $$\begin{array}{l} Mẍ+Cẋ+Kx=f+f_{r} \end{array}$$

where *f*_*r*_ and *f* are joints torques and the user-applied force, respectively. *M* denotes the mass matrix, *C* is the damping vector, and *K* contains the stiffness factors. The objective of controlling the manipulator is to minimize the error according to the desired TCP trajectory, i.e., *e* = *x*_*d*_ − *x*, which results in the following equation:

(10) $$\begin{array}{l} Më+Cė+Ke=f \end{array}$$

The controller should follow the desired trajectory in the presence of uncertainties, such as user-applied force. The position and velocity of joints can be controlled by defining the sliding mode error tracking, defined as Anvaripour et al. (2019):

(11) $$\begin{array}{l} r=ė+\alpha e \end{array}$$

Consequently, the controller equation would be

(12) $$\begin{array}{l} f=h\left(q,\dot{q},e,ė\right)-f_{r} \end{array}$$

where *h* is a function of joint and endpoints positions as well as the error between desired and actual positions. In many practical situation, the gravity is usually mechanically compensated, therefore, it is not considered in the dynamics differential equation.

The parameters of the controller should be adjusted such that robot reactions and TCP velocity are determined according to estimated human intentions, e.g., evasive motions in cases of unintended arm movements. In this regard, the robot controller should adjust the joint torques by finding an appropriate *h* function.

## 4. Incorporating an Estimation of Human Intentions in Planning Robot Reactions

This section explains how FMG data and robot dynamics are simultaneously used to enhance the collaboration scheme. The proposed approach is learning-based and task-specific: the desired trajectory for each task is pre-defined and followed during sample executions of that task. A recurrent neural network is trained using features extracted from collected FMG data and the robot dynamics, including endpoint and joint positions and velocities, in this phase. Using two sources of information, i.e., FMG and robot dynamics, improves the reliability of the approach, as discussed in Medina et al. (2017).

### 4.1. Proposed Recurrent Neural Network Scheme

For the purpose of this study, long short term memory (LSTM), an enhanced topology with less complexity compared to standard recurrent neural networks, was implemented (Srivastava et al., 2014). A standard LSTM can be defined as Yao et al. (2015):

(13) $$\begin{array}{l} \begin{array}{c} i_{t}=\sigma \left(W_{xi}x_{t}+W_{hi}h_{t-1}+W_{ci}c_{t-1}\right)\\ f_{t}=\sigma \left(W_{xf}x_{t}+W_{hf}h_{t-1}+W_{cf}c_{t-1}\right)\\ c_{t}=f_{t}c_{t-1}+i_{t}tanh\left(W_{x}c_{xt}+W_{hc}h_{t-1}\right)\\ o_{t}=\sigma \left(W_{xo}x_{t}+W_{ho}h_{t-1}+W_{co}c_{t}\right)\\ h_{t}=o_{t}tanh\left(c_{t}\right) \end{array} \end{array}$$

where *i*_*t*_, *f*_*t*_, and *o*_*t*_ are the input, forget, and output gates, respectively. Moreover, *x* and *c* are the input data and states, respectively. W denotes the matrix corresponding to each equation, and *h* is the output at the final time step/cell.

In the proposed approach, at each time step, selected features are extracted from FMG data and RD, *x*_*t*_ in (13), and a probability is assigned to each possible class of motions at the output of the LSTM, *h*_*t*_ in (13). Subsequently, the classification is achieved based on probability distribution. The feature vector,*c*_*t*_ in (13) is then passed to the next time step through the embedding layer.

**Figure 2:**
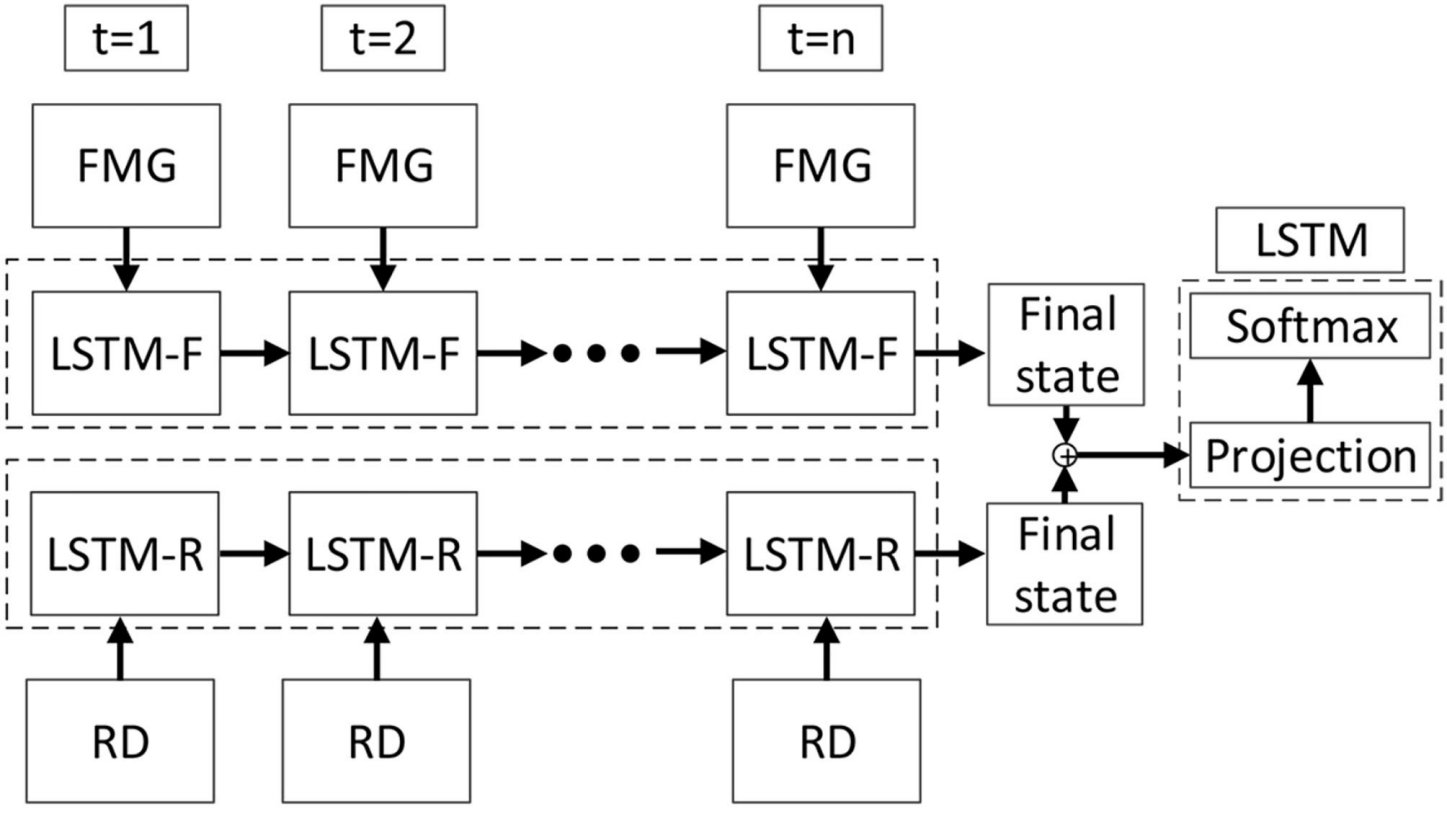
Schematic of the method proposed for estimating human intentions. The length of the LSTM cell is *n*. Features extracted from FMG and robot dynamics (RD) are inputs to the neural network at each time step.

Figure 2 shows the proposed LSTM topology that trains the system using features extracted from FMG data and RD. The information is passed to the fixed-length context vector of size *n*. The final state represents the probability distribution defining the vector of selected features. Following the proposed approach, at each time step *t* = *n*, data collected during *t* = 1, …, *n* are used by the network to estimate human intentions during this period.

**Figure 3:**
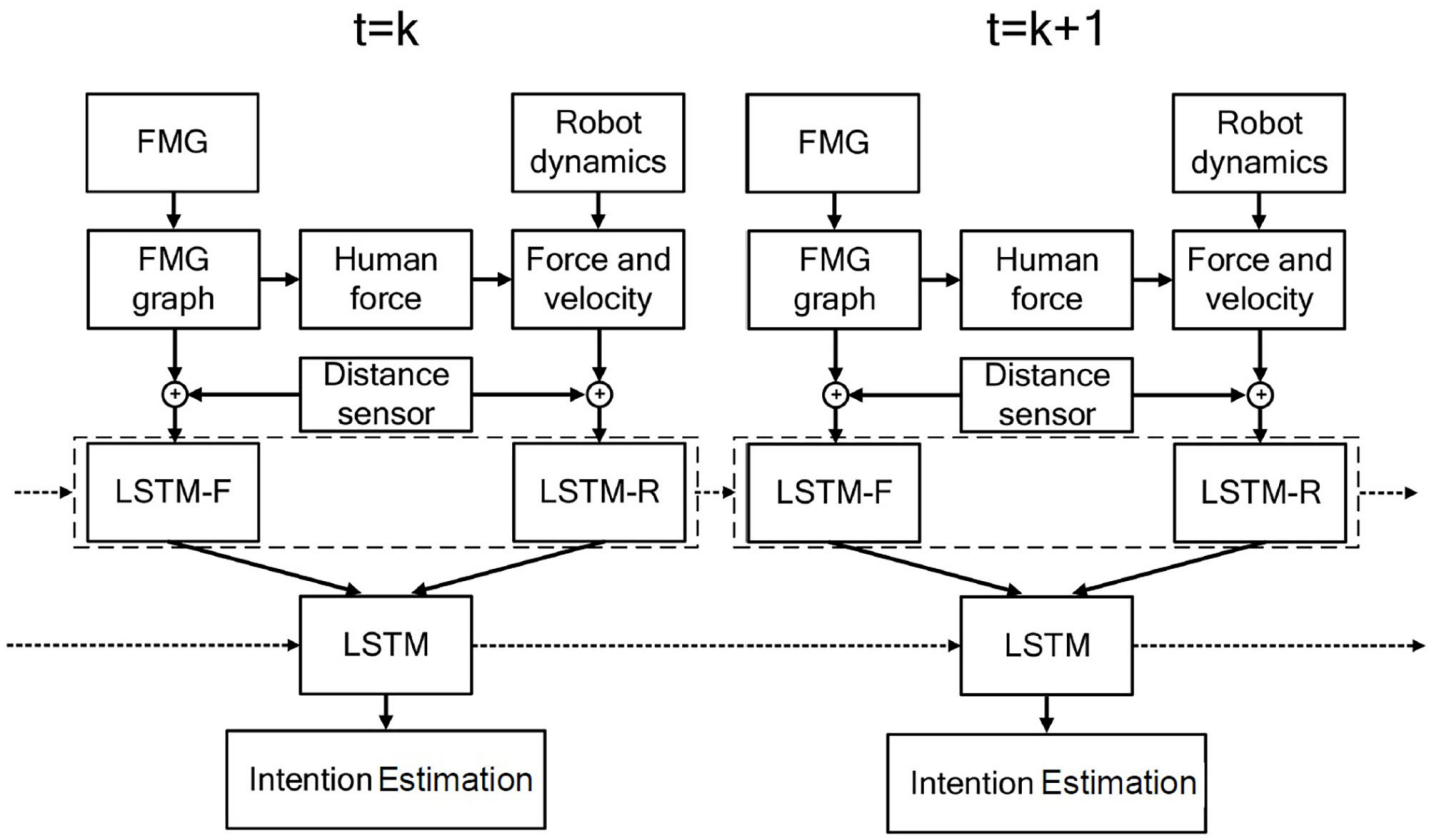
Block diagram of the information flow in two consecutive time steps.

Since in this study, two sources of information, i.e., FMG data and RD, are used to provide the required information about the human-robot interaction, two LSTM networks are formed: The LSTM-F network uses information from the FMG data collected from human forearm muscles, and the LSTM-R network uses information about the robot endpoint velocity and joint location. To form the feed-forward network, the two probability distributions obtained from these two networks are used as inputs to the hidden layer of the final LSTM (Figure 2), which applies the SoftMax method to estimate human intentions. To output such an estimation, *a*, a probability distribution over a set of possible intentions is obtained using *U*_*p*_ and *l*_*t*_:

(14) $$\begin{array}{l} P\left(a_{t+1}|a_{t},\left[F_{t},R_{t}\right]\right)=SoftMax\left(U_{p}\left[F_{t},R_{t}\right]+b\right) \end{array}$$

where *a* is the output estimated intention. *F*_*t*_ and *R*_*t*_ are the output vectors of LSTM-F and LSTM-R, respectively, that are considered as inputs to the SoftMax, and *U*_*p*_ and *b* are the parameters to be learned. Since the proposed strategy estimates whether a contact between the human and the robot is intentional or not, the result is a binary classification. In this case, the loss function for training the model is defined as

(15) $$\begin{array}{l} logP\left(a|l\right)\geq E\left[logP\left(a|l\right)\right]-KL\left[Q\left(F|a,R\right)||P\left(F|R\right)\right] \end{array}$$

where *l* is the vector of the concatenated *F* and *R*. The distribution *Q*(*F*|*a, l*) is an approximate posterior distribution, which aims to approximate the intractable true posterior distribution. The first term can be rewritten as ∑*logP*(*a*_*t*+1|*a*_*t*_, [*F*_*t*_, *R*_*t*_]_), which is calculated by SoftMax layer. The second term *KL*[*Q*(*F*|*a, l*)||*P*(*F*|*l*)], namely the KL term, is the Kullback-Leibler divergence which measures the non-symmetric difference between two probability distributions (i.e., *Q*(*F*|*a, l*) and *P*(*F*|*l*)) Krishnan et al. (2015). Figure 3 demonstrates the information flow in two consecutive time steps.

### 4.2. Planning the Robot Reaction

The RNN described in the previous subsection provides insight into human intentions. The next step is to plan the robot reaction such that the robot collaborates during intended movements to perform the shared tasks but performs evasive motions or activates brakes in case of unintended movements.

The distance sensor mounted on the robot arm can provide information about the distance between the robot and the human worker as well as the direction of potential collision. Denoting the distance sensor reading by $\vec{dist}$, an effective way to derive the joint movement is by utilizing the impedance approach with interpreting $f\vec{dist}$ as virtual force at the *i*th joint of the robot. In this case, $f\vec{dist}$ is achieved by (12). As discussed in Chiaverini (2008) and Avanzini et al. (2014), the torque vector can be calculated from

(16) $$\begin{array}{l} \tau =\overset{n}{\underset{i=1}{\sum }}J_{i}^{T}.f{\vec{dist}}_{i} \end{array}$$

where *J*_*i*_ corresponds to the Jacobian matrix of the *i*th Denavit-Hartenberg frame position corresponding to the *i*th link of the robot. The resulting ***τ*** represents the control action to be applied to the robot. By taking into account the information provided by the vector of ${\vec{dist}}_{i}$, the evasive motion that moves the robot away from the human worker can be determined. The joint's evasive motion can be defined by a mass-damper model Avanzini et al. (2014) and expressed by joint velocity as

(17) $$\begin{array}{l} {\dot{q}}_{ev}={\left(M.s+C\right)}^{-1}.\tau \end{array}$$

where *s* indicates the Laplace domain, and *M* > 0 and *C* > 0 are the mass matrix and damping vector, respectively.

A reward function, denoted by *R*, was used to establish how the FMG and RD probability distributions were used to control the robot movement. This reward function was defined as

(18) $$\begin{array}{l} R=\overset{n}{\underset{j=1}{\sum }}{\dot{q}}_{ev}^{j}P\left(LSTM-F\right)P\left(LSTM-R,h_{j}\right) \end{array}$$

where *P*(*LSTM* − *F*) is the resulting probability distribution at the final state of the LSTMF memory cell, *P*(*LSTM* − *R, h*) is the extracted probability distribution at the final state of the LSTM-R considering the robot controller state, and *h*_*j*_ is the state of the joint *j* that is estimated by the controller throughout the task operation. By evaluating the robot endpoint position and speed, possible collisions or dangerous actions can be avoided when a human uses his hand to interact with the robot. The final step LSTM using the Softmax method assigns probabilities to different anticipated actions, categorized as intended and unintended.

## 5. Experimental Evaluation

### 5.1. Experimental Setup

To evaluate the proposed approach, a YUMI manipulator robot (ABB Robotics, Switzerland) was used. This robot has two 7-DOF arms with grippers, as shown in Figure 5A. The controller measures and controls arm movements in Cartesian coordinates. The robot parameters during the operation are recorded by Robot Studio software developed by the ABB company. The maximum tolerable force, *f*_*max*_, for this robot is 20 N Robotics (2015). To mimic the passive stiffness in human forearm muscles, the minimum force was set to *f*_*min*_ = 0.01 N. The position of the end-point and joints was predetermined in the controller. A laptop equipped with Robot Studio, MATLAB (MathWorks, Natick, MA), and TinyOS software was used as the base station and communicated with the robot via an Ethernet cable.

**Figure 4:**
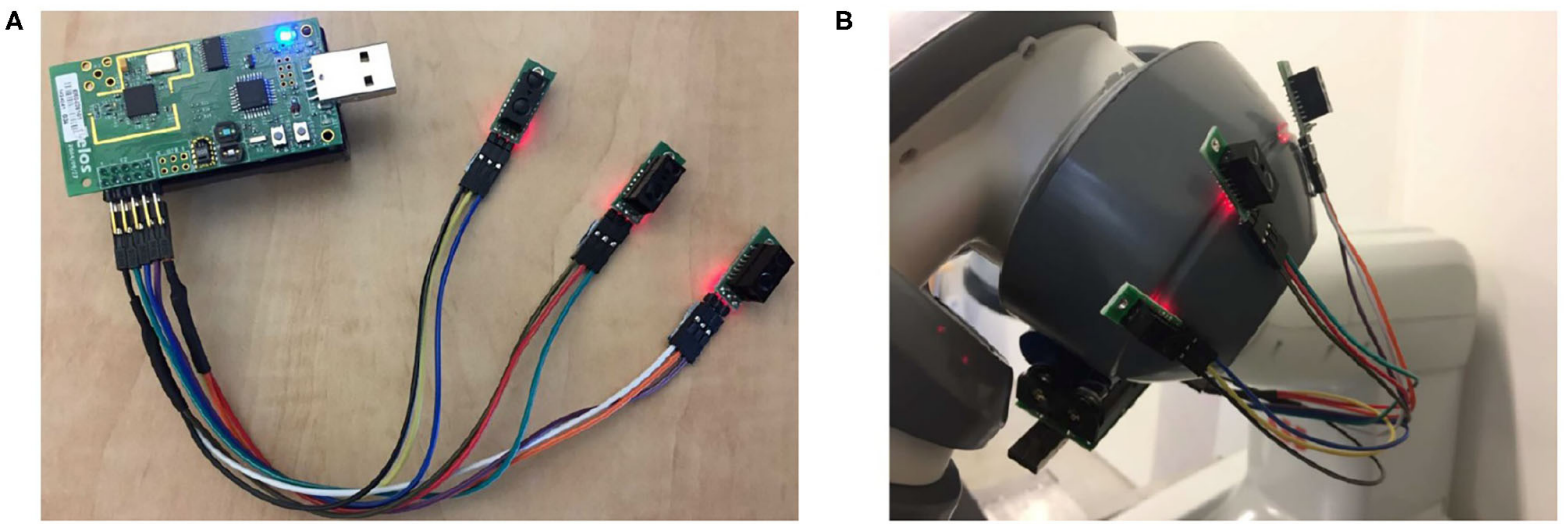
Distance sensors used to detect obstacles within a 5-cm range. **(A)** sensors mounted on the TelosB wireless mote, **(B)** sensors affixed to the robot joints.

Three distance sensors (Sharp GP2Y0D805Z0F) were mounted on the TelosB wireless mote and were affixed to the second and third joint of the left robotic arm (Figure 4). These sensors can detect obstacles in the 5-cm distance, and their data were sent to the base station through the mote using wireless communication.

Considering that eight FSRs were used in the FMG band (Figure 1), the FMG graph (Equation 4) had 8 vertices and 28 edges. The FMG data captured from the human forearm were used to train the LSTM-F, and recorded joints positions and velocities were applied to train the LSTM-R. The LSTM was tested with a sigmoid activation function for cells 1–9, and two sets of features, i.e., FMG data and robot dynamics, were considered in the proposed multimodal topology. Since in real-world applications, different collaboration scenarios might occur, the LSTM-R network was trained for each scenario separately, as explained in the next subsection.

### 5.2. Training the LSTM Network

**Figure 5:**
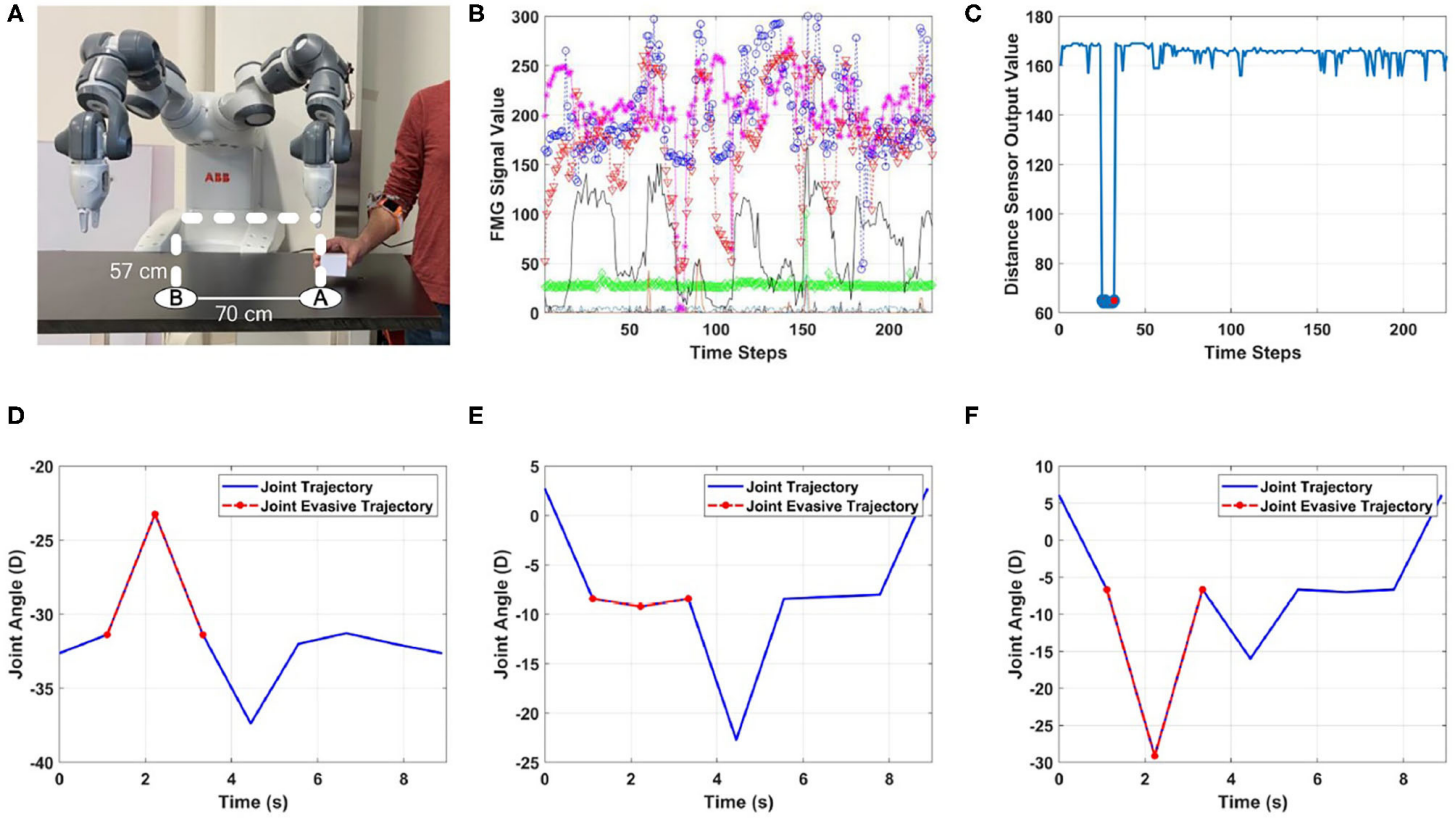
Scenario 1: unintentional human-robot contact when distance sensors are used. **(A)** Human works to the side of the robot to place the packed box at location A, **(B)** FMG values (output of each sensor is shown with a different color), **(C)** output of the distance sensor, **(D–F)** evasive motion trajectory of joints 2, 3, and 4.

Ten healthy adults (31.7 ± 6 years old; 4 females and 6 males) with limited prior experience in working alongside the robot consented to participate in this study. The participants were asked to wear the FMG band around their right forearm and to touch the robot manipulator 50 times following a self-chosen trajectory, while the robot moved along a predefined trajectory to relocate a part (see Figure 5A). The LabView platform was used for data acquisition, and FMG data were collected with a sampling frequency of 10 Hz. The corresponding data collection session for each participant was about 100 s. Using a sliding window of 10 samples with a 5-s overlap resulted in 10,000 data points per participant, and therefore, 100,000 data points for all 10 participants. The model was developed using 10-fold cross-validation, and its performance was further evaluated by asking five participants to return for a second data collection session similar to the first one. These individuals were a subset of the ten participants who provided data for the training phase and were selected based on their availability to attend a second data collection session. The goal was to replicate the situation in which the same worker wears the FMG band during different working days. It is also worth noting that the FMG band might not be worn on the exact same location on the forearm everyday. Testing the proposed model in such a situation demonstrated how the model dealt with the effects of slight relocation of the band.

### 5.3. Sample Collaboration Scenarios

In this section, two human-robot cooperation scenarios that might arise during packing and moving of objects are presented.

The first scenario considers the case in which the human works in proximity of the left robotic arm and helps packing and moving of small boxes. In this case, intentional contact is defined as the situation in which the worker touches the robot arm with a plan, e.g., to manually adjust it. However, when the worker accidentally gets too close to/touches the robot arm, an unintentional contact happens. In the intentional contact, the robot would stop working and release brakes to allow the worker to manually readjust the joints configuration. When an unintentional contact is detected, the robot controller should either stop working to prevent injury to the worker or adjust joint positions to prevent collision while continuing to the task.

The second scenario deals with the case in which the human is working in front of the robot. Such a situation might arise when the worker should manually adjust the endpoint location during the process. In this case, the human intention should be estimated and distinguished from random contacts which might lead to a collision. To cooperate with the human worker, the controller should release the joint related to the gripper of the robot so that the gripper location can be manually adjusted. If the controller detects an unintended contact, it would stop the robot immediately to prevent possible injury to the worker's hand.

Table 1 summarizes how the robot reacts to intended or unintended contacts with or without using distance sensors in each scenario.

**Table 1:** Robot reactions defined to provide safe human-robot interaction.

Intended contact
	With distance sensors	Stop
		Release brakes
	Without distance sensors	Stop
		Release brakes
Unintended contact
	With distance sensors	Evasive motion
		Slow down, brake
	Without distance sensors	Stop
		Slow down, brake

#### 5.3.1. Scenario 1: Human Works Next to the Robot

In this experiment, the participant packed a small box and placed it at marked location (A) to be moved by the robot to location (B). The human stayed close to the left side of the robot as shown in Figure 5A. In this scenario, the performance of the proposed method was evaluated in two situations.

*The first situation* dealt with the case in which data from distance sensors were considered, and human intentions were estimated using FMG data. If the contact was classified as intended, the robot controller decreased the TCP velocity and released the brakes. In case of unintended contacts, the controller planned evasive joint angles to avoid collision.

In *the second situation*, it was assumed that data from distance sensors were not available. The human intention was still estimated using the FMG data, however, the robot reaction to a detected contact was to decrease the TCP velocity and apply brakes.

To train the proposed RNN algorithm, the worker donned the FMG band on their forearm, packed three boxes with different weights (Table 2), and placed them on the marked location. This procedure was repeated 10 times. Subsequently, the participant was asked to touch the robotic arm 30 times with the FMG band-wearing hand. Consequently, a total of 60 sequences of data, i.e., 30 sequences corresponding to packing and placing of parts and 30 sequences of human-robot contact, were collected and used to train the LSTM-F network. To train the LSTM-R, joint angles in Cartesian coordinates, TCP velocity, and brakes status were included in the controller and were monitored while the robot was operating in the presence of the human worker.

**Table 2:** Specifications of parts used in the experiments.

**Parts**	**1**	**2**	**3**
Size (cm)	3 × 3 × 1.5	5 × 3 × 2	6 × 5 × 4
Weight (g)	100	250	500

To evaluate the trained model, the human worker performed packing and placing of boxes for 5 min, during which they intentionally and unintentionally touched the robotic arm. Figure 5 shows the results for a case in which distance sensors were used, and worker's shoulder came very close to the robot arm when picking up a part. The distance sensor on Joint3 of the robot arm, which was closer to the worker, detected the obstacle (Figure 5C), and the controller initiated the evasive motion of joints to prevent collision (Figures 5D–F). For simplicity, only trajectories of joints 2, 3, and 4 of the robot arm are shown. For the sake of clarity, in Figures 5D–F, the evasive motions and joints angles are only shown during 8 s of task operation, corresponding to when the robot moves down to pick up the part and return to its initial position.

**Figure 6:**
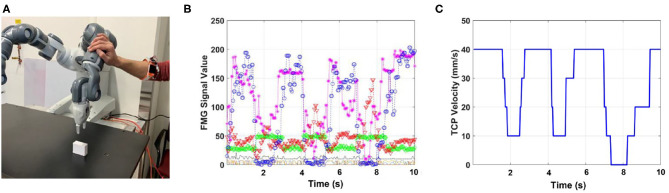
Scenario 1: intentional human-robot contact when distance sensors are not used. **(A)** Human intentionally manipulates the robotic arm, **(B)** FMG values (output of each sensor is shown with a different color), **(C)** TCP velocity reduced when intended contact is detected. The robot stops when the worker readjusts joint configurations during the third interaction.

In the second part of the experiment, it was assumed that distance sensors were not available. The human worker intentionally touched the robotic arm three times during performing of the task. Human intentions were estimated based on FMG data (Figure 6A). During the first two contacts, the controller reduced the velocity of TCP and released the brakes. However, as the worker readjusted the configuration of robotic joints during the third intended contact, the controller stopped the robot and released the brakes (Figures 6B,C).

The proposed method combines data from both FMG sensors and robot dynamics to make a decision about the nature of contact. To compare it performance with that of using either FMG data or robot dynamics, F1 factor was calculated as a measure of intention recognition performance (Schydlo et al., 2018). This factor was obtained by 3-fold cross-validation, and the average is reported. F1 factor is defined as Schydlo et al. (2018)

(19) $$\begin{array}{l} F1=\frac{2\times TruePositive}{2\times TruePositive+FalseNegative+FalsePositive} \end{array}$$

**Figure 7:**
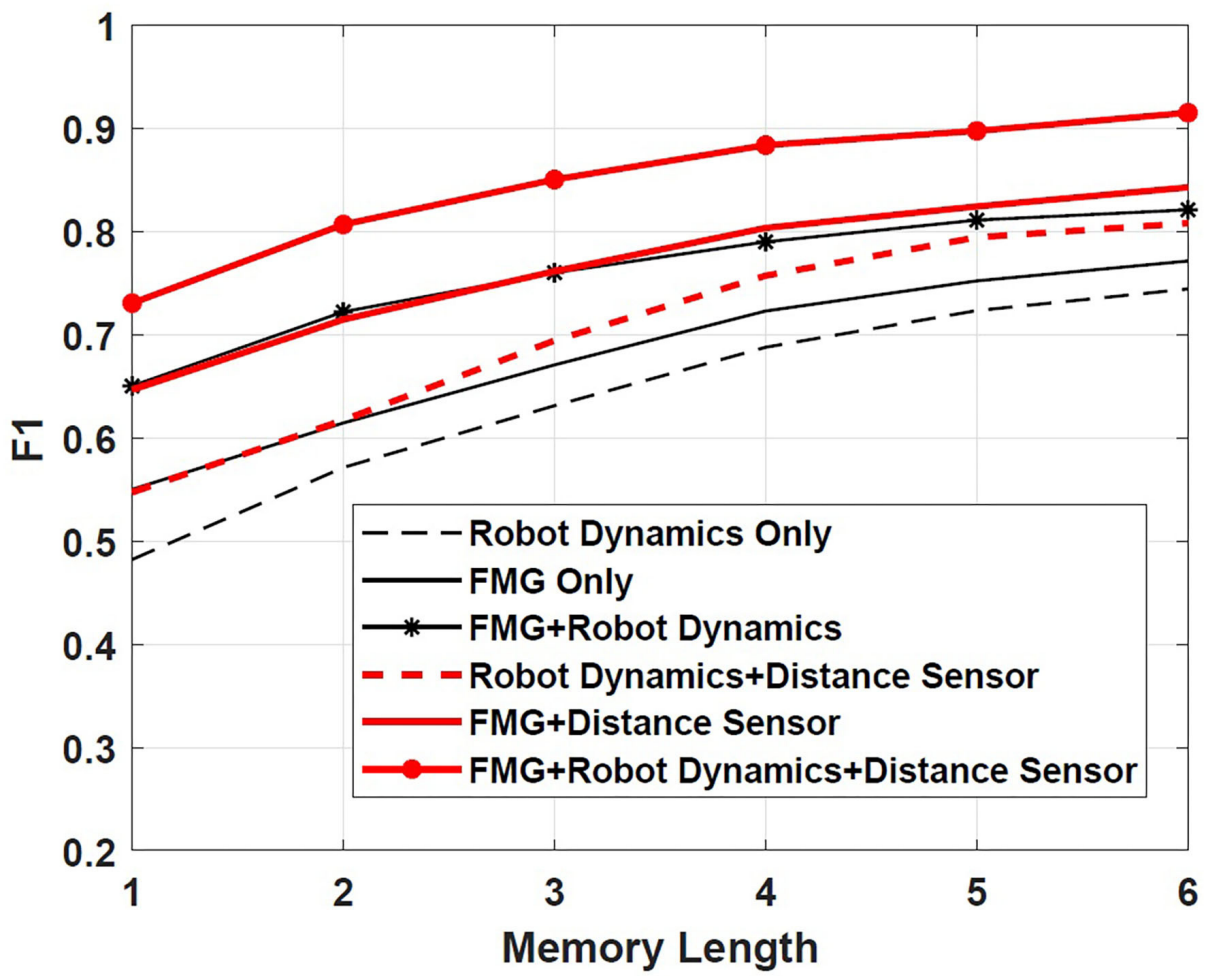
Scenario 1: effect of the length of LSTM cells on F1 factor.

By this definition, 0 ≤ F1 ≤ 1, where *F*1 = 1 denotes flawless performance of the recognition algorithm. Figure 7 shows how the length of LSTMs affects the intention prediction and the subsequent robot reaction. It is observed that increasing the number of LSTM memory cells results in an F1 factor closer to 1. Moreover, as shown in Figure 7, the proposed multi-modal approach considering both FMG data and robot dynamics boosts the performance in comparison with cases in which these information sources were separately applied. Using distance sensors to detect objects closer than 5 cm to the robotic arm further improves the F1 factor and has a positive effect on estimating intentions. Considering that the sampling period was 0.04 s and a window width of 10 samples with a 5-sample overlap between windows was chosen, the total time required for the intention prediction process using three memory cells was: 3 × (10 × 0.04) − 2 × (5 × 0.04) or 0.8 s.

#### 5.3.2. Scenario 2: Human Works in Front of the Robot

**Figure 8:**
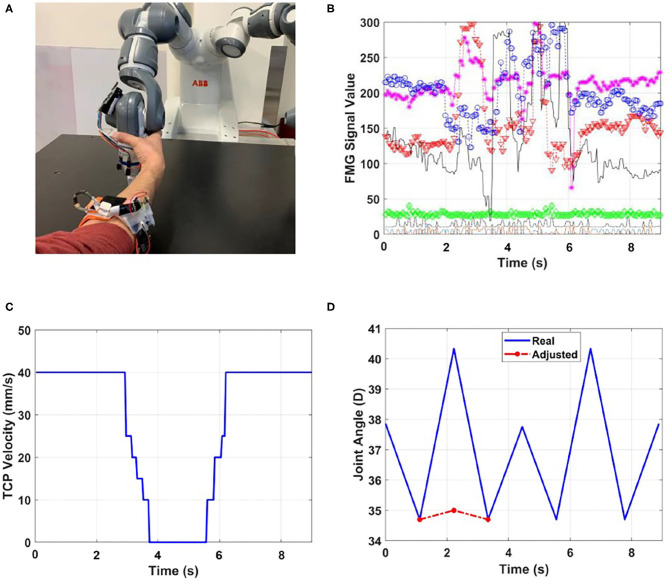
Scenario 2: intentional human-robot contact when distance sensors are not used. **(A)** Human intentionally adjusts the position of TCP, **(B)** FMG values (output of each sensor is shown with a different color), **(C)** TCP velocity, **(D)** adjusted joint angle considering human manipulation.

In this part of the experiment, the human worker performed the collaborative task while standing in front of the robot. Similar to Scenario 1, the worker donned the FMG band on their right forearm, and the proposed approach was tested in two different modes of using or not using the data from distance sensors. In this scenario, the right arm of the robot was used to interact with the human worker, and the distance sensors were affixed to joints 5 and 6 which are close to the robot gripper (Figure 8A).

Figure 8 shows results for the case in which the worker manipulated the robotic arm to adjust the position of its endpoint or the TCP, and the data from distance sensors were not available. It is seen in Figure 8 that the intended contact was correctly detected, and the controller stopped the robot and released the brakes to cooperate with the human.

**Figure 9:**
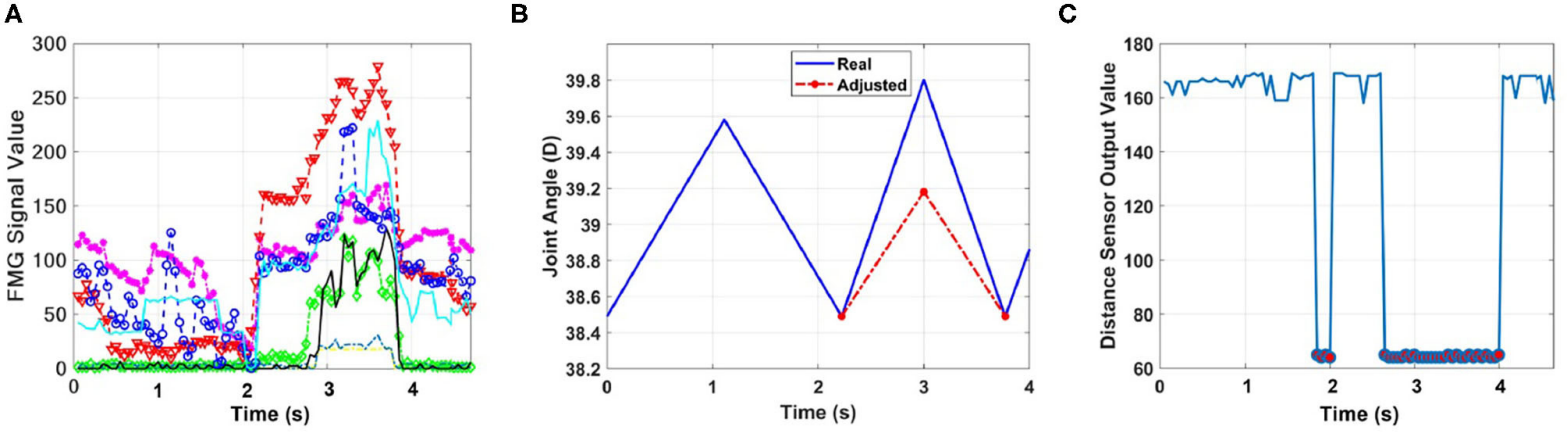
Scenario 2: intentional human-robot contact when distance sensors are used. **(A)** FMG values (output of each sensor is shown with a different color), **(B)** the adjusted joint angle, **(C)** output of the distance sensor.

When distance sensors were used to report obstacles within the 5-cm range, the controller stopped the robot to avoid collision when intended contact was detected. Figure 9 shows the results for the case in which the worker modified the TCP position. The change in the output of the distance sensor (Figure 9C) indicated the contact, and consequently, the controller adjusted the angle of joint6 to collaborate with the human (Figure 9B).

**Figure 10:**
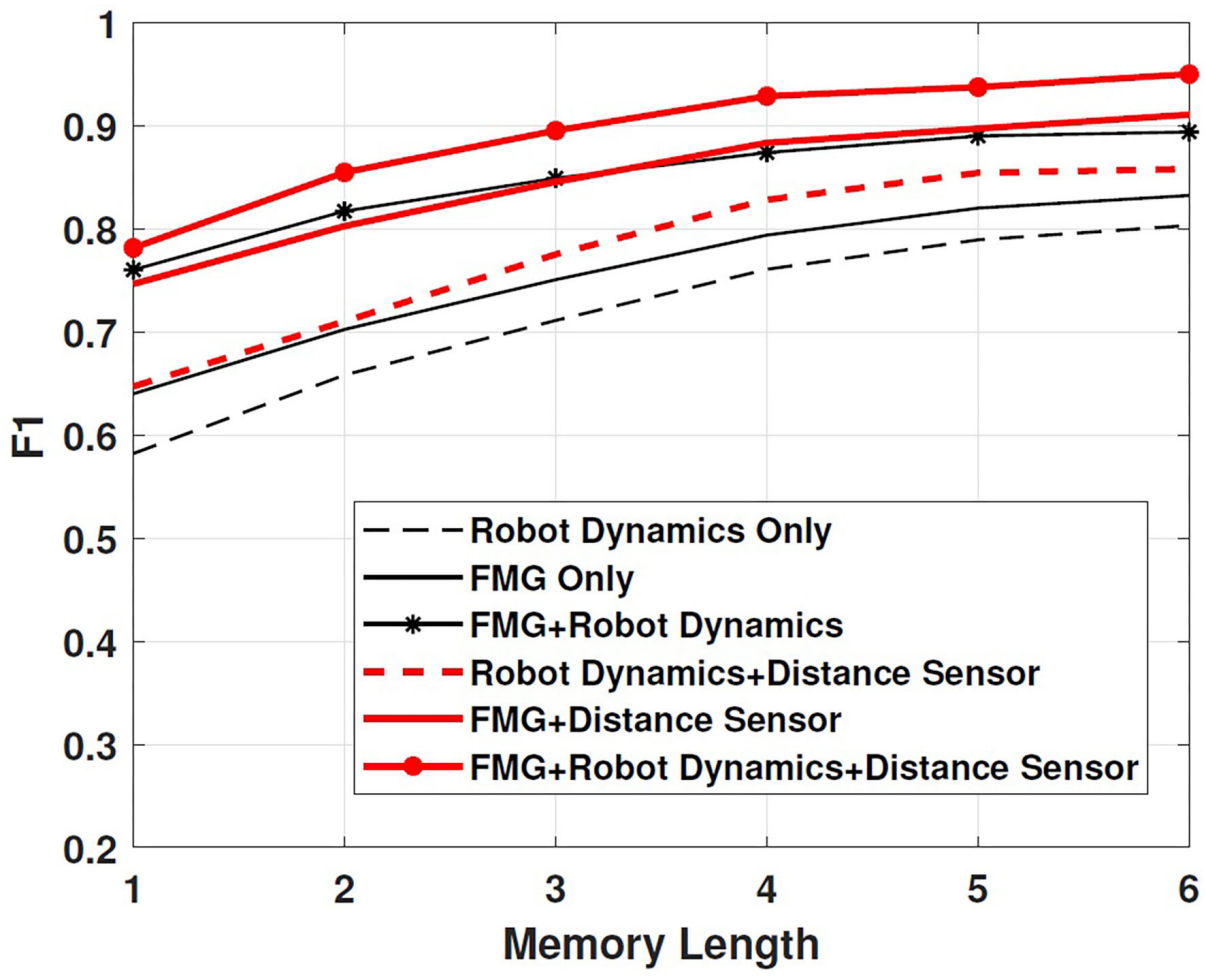
Scenario 2: effect of the length of LSTM cells on F1 factor.

To assess if considering information from both FMG data and robot dynamics improved the performance, F1 factor is calculated in this scenario as well. Figure 10 shows that the proposed multi-modal approach considering both FMG and robot dynamics data along with the distance sensors results in the most reliable intention estimation. This result is consistent with that obtained in Scenario 1.

## 6. Conclusions

This paper presented a neural network-based method to incorporate human intentions in human-robot collaboration scenarios. In this regard, force myography data, collected from the human forearm, and robot dynamics were used to train a recurrent neural network to estimate human intentions. A control algorithm was then implemented to plan appropriate robot reactions considering the outcome of this estimation. The performance of the proposed method was evaluated experimentally, and successful human-robot collaboration during two practical scenarios was demonstrated. It was also shown that having a measure of distance between the human worker and the robot further boosts the performance. Moreover, the experimental evaluation showed that the proposed approach could estimate human intentions in <1 s. The results of this study show that a system incorporating human muscle information (FMG data), robot dynamics, and environment factors (the distance between the human and the robot) could provide necessary tools for improved and flexible human-robot collaboration.

Instrumenting the robot with new sensor technologies, such as passive tactile sensors, to provide information about the distance between the human user and the robotic arm, and implementing more advanced machine learning techniques to increase estimation accuracy by using data from multiple sensing sources are next steps in this research.

## Data Availability Statement

The raw data supporting the conclusions of this article will be made available by the authors, without undue reservation.

## Ethics Statement

The studies involving human participants were reviewed and approved by University of Windsor Research Ethics Board (REB). The patients/participants provided their written informed consent to participate in this study.

## Author Contributions

MA, CM, and MS conceived and designed the experiments. MA implemented the experimental setup, collected, and analyzed the data. MA and MK wrote the manuscript. All authors contributed to the article and approved the submitted version.

## Conflict of Interest

CM and members of his research team have a vested interest in commercializing the FMG technology tested in this study, if it is proven to be successful, and may benefit financially from its potential commercialization. The remaining authors declare that the research was conducted in the absence of any commercial or financial relationships that could be construed as a potential conflict of interest.
